# Cell-Extrinsic Defective Lymphocyte Development in *Lmna^-/-^* Mice

**DOI:** 10.1371/journal.pone.0010127

**Published:** 2010-04-12

**Authors:** J. Scott Hale, Richard L. Frock, Sara A. Mamman, Pamela J. Fink, Brian K. Kennedy

**Affiliations:** 1 Department of Immunology, University of Washington, Seattle, Washington, United States of America; 2 Department of Biochemistry, University of Washington, Seattle, Washington, United States of America; New York University, United States of America

## Abstract

**Background:**

Mutations in the *LMNA* gene, which encodes all A-type lamins, result in a variety of human diseases termed laminopathies. *Lmna^-/-^* mice appear normal at birth but become runted as early as 2 weeks of age and develop multiple tissue defects that mimic some aspects of human laminopathies. *Lmna^-/-^* mice also display smaller spleens and thymuses. In this study, we investigated whether altered lymphoid organ sizes are correlated with specific defects in lymphocyte development.

**Principal Findings:**

*Lmna^-/-^* mice displayed severe age-dependent defects in T and B cell development which coincided with runting. *Lmna^-/-^* bone marrow reconstituted normal T and B cell development in irradiated wild-type recipients, driving generation of functional and self-MHC restricted CD4^+^ and CD8^+^ T cells. Transplantation of *Lmna^-/-^* neonatal thymus lobes into syngeneic wild-type recipients resulted in good engraftment of thymic tissue and normal thymocyte development.

**Conclusions:**

Collectively, these data demonstrate that the severe defects in lymphocyte development that characterize *Lmna^-/-^* mice do not result directly from the loss of A-type lamin function in lymphocytes or thymic stroma. Instead, the immune defects in *Lmna* ^-/-^ mice likely reflect indirect damage, perhaps resulting from prolonged stress due to the striated muscle dystrophies that occur in these mice.

## Introduction

Nuclear lamins are intermediate filament proteins and have roles in various nuclear processes, including DNA replication, chromatin organization, and gene transcription [1]–[4]. One gene, *LMNA*, encodes the A-type nuclear lamins, which produce the predominant A-type lamin proteins A and C. Mutations in *LMNA* are associated with more than 13 different tissue-specific diseases, collectively termed laminopathies, which include muscular dystrophies, cardiomyopathies, and more recently, a series of progeroid diseases that resemble some aspects of premature aging (for review, see Cohen et al.) [5]. *Lmna^-/-^* mice are characterized by a variety of tissue-specific defects consistent with those observed in human laminopathies, but additionally display growth defects as early as 2 weeks of age, reduced thymus and spleen size, defective spermatogenesis, and death by 6–8 weeks of age [6], [7].

A-type lamins have limited expression in the hematopoietic system [8]. Previous studies describing the presence of lamin A/C proteins in lymphocyte lineages are mixed, but largely suggest limited to no presence of A-type lamin proteins in early stages of B and T cell development, with increasing protein abundance in mature B and T cells and stimulated lymphocytes [9]–[13]. Furthermore, A-type lamin proteins are found only in a minority of cells from the bone marrow, thymus, spleen, and lymph nodes, which may represent cell types of the stroma such as epithelial cells, pericytes, inflammatory cells, fibroblasts, endothelial cells, and smooth muscle cells [8], [13]–[15].

Although no immune disease to date has been linked to mutations in *LMNA*, *Lmna^-/-^* mice display reduced thymus and spleen size, suggesting a potential role for A-type lamins in the postnatal development and/or homeostasis of lymphocyte lineages. Here, we investigate the reduced thymus and spleen size in *Lmna^-/-^* mice and identify a progressive, age-dependent impairment in T and B cell development. These defects are not cell-autonomous, because transplanted *Lmna^-/-^* bone marrow can reconstitute irradiated recipient *Lmna^+/+^* immune tissues. Transplanted *Lmna* ^-/-^ thymus lobes show good engraftment in wild-type hosts and drive normal thymocyte development. Therefore, the altered immune cell development is not specific to loss of *Lmna* expression in developing lymphocytes or thymic epithelial cells, but likely an indirect effect of loss of lamin expression in other non-lymphoid tissues.

## Methods

### Mice

*Lmna^+/-^* mice were obtained from Colin Stewart (Institute of Medical Biology, Immunos Singapore) [7] and sibling mating was used to generate *Lmna^+/+^* and *Lmna^-/-^* mice. This line was also backcrossed to C57BL/6J (B6) mice for 9 additional generations and the resulting mice, B6.129S1(Cg)-Lmna^tm1Stw^/BkknJ, were used for thymus transplantation studies. B6.SJL (B6.SJL-*Ptprc^a^Pepc^b^/BoyJ*) Ly5.1^+^ and C57BL/6J Ly5.2^+^ mice were crossed to generate F1 Ly5.1^+^Ly5.2^+^ mice used as recipients in bone marrow chimera experiments. Mice were bred and maintained under specific-pathogen free conditions. All experiments were conducted in accordance with the review board of the University of Washington Institutional Animal Care and Use Committee, who approved this study.

### Antibodies and flow cytometry

Single-cell suspensions were prepared from thymus, spleen, bone marrow, and a pool of brachial, axillary, and inguinal lymph nodes. Red blood cells were removed from spleen cells by water lysis. For flow cytometry, Fc receptors were blocked using anti-CD16/32 (2.4G2; BD Pharmingen). Cells were surface stained in BSS containing 1% BSA with FITC, phycoerythrin- (PE), Peridinin-Chlorophyll-Protein-Cy5.5, PE-Cy7, allophycocyanin- (APC), and APC-AlexaFluor 750 flourochrome-conjugated antibodies and, in some cases, biotinylated antibodies followed by FITC- or APC-conjugated streptavidin. Antibodies were purchased from BD Pharmingen or eBioscience and included monoclonal antibodies recognizing mouse CD4 (RM4-5), CD8α (53–6.7), CD19 (1D3), CD25 (PC61), CD44 (IM7), CD45R/B220 (RA3-6B2), CD62L (MEL-14), CD69 (H1.2F3), panTCRβ (H57-597.13), IgM (II/41), and IgD (11–26c). For intracellular cytokine staining, splenocytes were incubated for 5 hours at 37°C in RP10 (RPMI with 10% FBS, 10 mM HEPES, 4 mM L-Glutamine, 100 U/ml penicilin, 100 µg/mL streptomycin, 50 µM β-mercaptoethanol, and 50 µg/mL Gentamycin) in the presence of GolgiPlug (BD Pharmingen) either in the absence or the presence of 1 µg/mL GP_61–80_ I-A^b^-restricted peptide (GLKGPDIYKGVYQFKSVEFD) for detection of Lymphocytic Choriomeningitis Virus (LCMV)-specific CD4^+^ T cells or 0.1 µg/mL GP_33–41_ H-2D^b^-restricted peptide (KAVYNFATC) (Invitrogen) for detection of LCMV-specific CD8^+^ T cells [16]. Cells were surface-stained first, and then stained for intracellular cytokines with APC-conjugated anti-IFNγ (XMG1.2) and PE-conjugated anti-IL-2 (JES6-54H4) or anti-TNFα (MP6-XT22), using the BD Cytofix/Cytoperm kit and protocol. Flow cytometry data were collected using a FACS Canto (Becton Dickenson) and analyzed with FlowJo software (Treestar; Ashland, OR).

### Bone marrow chimeras

Single-cell suspensions of hind leg bone marrow from *Lmna* ^+/+^ and *Lmna* ^-/-^ mice were T cell- depleted by staining with PE-conjugated anti-CD4 and anti-CD8 followed by removal of PE-labeled cells using the EasySep PE selection separation kit (Stem Cell Technologies; Vancouver, Canada). Alternatively, T cells were depleted using anti-Thy1 (13.4.6), anti-CD4 (RL172), and anti-CD8 (3.168.8) followed by lysis using rabbit complement (Cedarlane Laboratories Limited; Hornby, Canada). Sex-matched recipient B6 Ly5.1^+^Ly5.2^+^ mice were irradiated with 1000 rads 6–8 hours prior to bone marrow injection, and maintained on antibiotic water (polymixin B sulfate and neomycin sulfate from Invitrogen), for 2 days prior to 2 weeks following irradiation. Recipient mice were injected with 3−5×10^6^ T cell-depleted bone marrow cells into the lateral tail vein.

### LCMV infection

Mice were infected by intraperitoneal injection with 2×10^5^ PFU of LCMV Armstrong (provided by M.K. Kaja; University of Washington). Spleens were harvested 8 days later for analysis of LCMV-specific CD4^+^ and CD8^+^ T cells by intracellular cytokine staining and flow cytometry.

### Thymic lobe transplantation

Thymic lobes from 1-day-old *Lmna^+/+^* and *Lmna^-/-^* neonates were transplanted into 10- to 16-week-old female *Lmna^+/+^* host mice by engraftment underneath the kidney capsule. Briefly, 2 days prior through 3 days after surgery, host mice were given medicated water (ibuprofen 0.2 mg/mL). Neonatal thymic lobes were placed on sterile nitrocellulose pads and suspended at the culture medium interface to ensure high oxygen transfer and cell survival. Thymuses were cultured at 37°C, 5% CO_2_ for less than 24 hours prior to transplantation while genotypes of extracted neonatal thymuses were being determined. The culture medium consisted of DMEM, 10% fetal calf serum, 1 mM sodium pyruvate, 2 mM L-glutamine, 100 U/mL penicillin, 100 µg/mL streptomycin, 0.1 mM non-essential amino acids, 0.3% sodium bicarbonate, 25 mM HEPES, and 50 µM β-mercaptoethanol. Host mice were anesthetized with ketamine and xylazine (130 mg/kg and 8.8 mg/kg respectively) and a laparotomy was performed after hair removal and sterilization of the surgical area. Thymic lobes were transferred underneath the left kidney capsule. Host mice were then sutured and monitored until full recovery from anesthesia. Transplanted thymuses were allowed to engraft for 6 weeks prior to analysis.

## Results

### Runting of *Lmna* ^-/-^ mice is accompanied by progressive thymic and splenic atrophy

**Figure 1:**
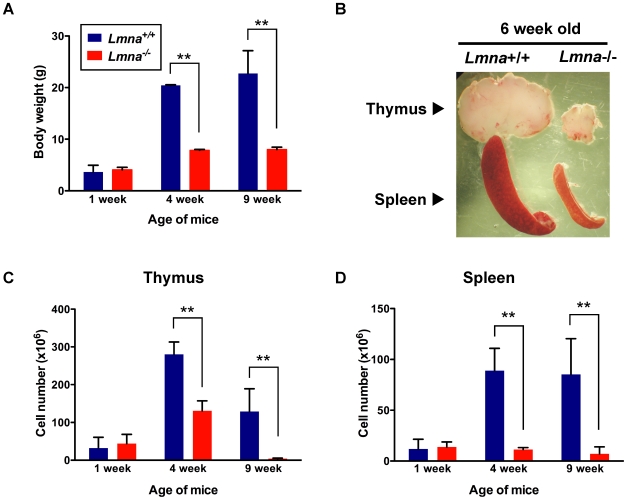
Age-dependent progressive decrease in body weight and spleen and thymus size in *Lmna* ^-/-^ mice. Body weight and splenic and thymic cellularity from *Lmna^+/+^* and *Lmna^-/-^* mice were determined at 1, 4, and 9 weeks of age. A. Body weight of mice from indicated age groups. B. Representative thymus and spleen from 6-week-old mice. C. Absolute numbers of thymocytes from mice of the indicated age groups. D. Absolute numbers of splenocytes from mice of the indicated age groups. Bars show the mean value with error bars indicating the standard deviation (*Lmna^+/+^* N = 3; *Lmna^-/-^* N = 3 for each indicated age group). P values were calculated using an unpaired two-tailed Student's t test (** p<0.001).

It has been previously reported that *Lmna* ^-/-^ have mice small lymphoid organs, including the spleen and thymus [7]. We analyzed lymphoid organs from *Lmna* ^-/-^ mice to better understand the effects of *Lmna* deficiency on the immune system. Neonatal *Lmna* ^-/-^ mice were indistinguishable from *Lmna* ^+/+^ littermates 1 week after birth. By 4 weeks of age and older, *Lmna* ^-/-^ mice were severely runted compared to *Lmna* ^+/+^ mice (Figure 1A). The thymus and spleen from 6-week old *Lmna* ^-/-^ mice were reduced in size (Figure 1B). We observed that while thymic cellularity in neonatal *Lmna* ^+/+^ and *Lmna* ^-/-^ mice was similar, 4-week old *Lmna* ^-/-^ mice displayed decreased thymic cellularity compared to *Lmna* ^+/+^ littermates that became more striking with age (Figure 1C). Similarly, splenic cellularity was unaffected in neonatal *Lmna* ^-/-^ mice, but was severely reduced at 4 and 9 weeks of age (Figure 1D). Thymic and splenic cellularity were reduced even when normalized to the reduced body weight of *Lmna* ^-/-^ mice (Figure S1).

### Defective thymocyte maturation in the atrophic *Lmna* ^-/-^ thymus

**Figure 2:**
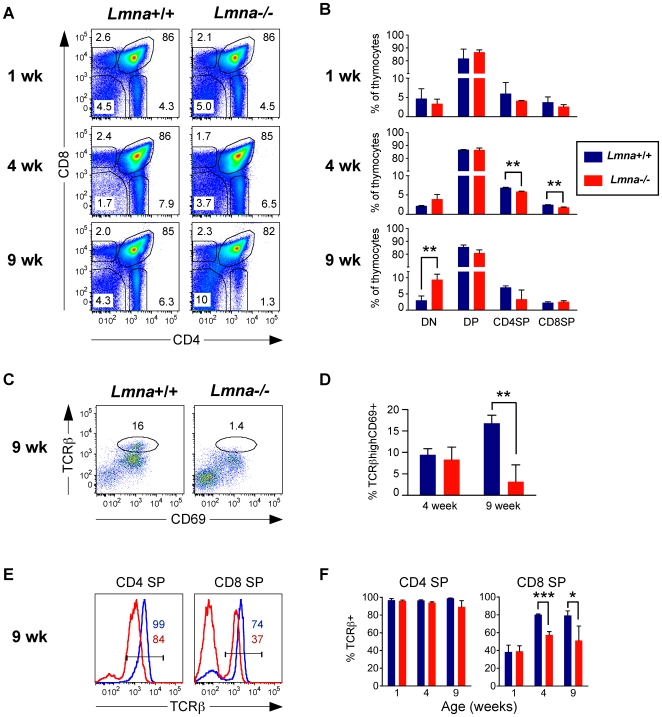
*Lmna* ^-/-^ mice display an age-dependent defect in thymic T cell development. Thymocytes were stained for CD4, CD8, CD69, and panTCRβ surface expression. A. Representative flow cytometry plots show CD4/CD8 analysis of live-gated thymocytes. B. Charts show the percent of thymocytes that are DN, DP, CD4 SP, and CD8 SP for each indicated age group compiled from all mice analyzed. C. CD69/TCRβ analysis of CD4^low^CD8^low^ gated DP dull cells from 9-week-old mice with a gate indicating the percent of post-selection TCRβ^high^CD69^+^ cells. D. The percent of CD4^low^CD8^low^ DP dull cells that are TCRβ^high^CD69^+^ for 4- and 9-week-old mice. E. Histograms of gated CD4 SP and CD8 SP cells showing TCRβ expression. Colored numbers (Blue: *Lmna* ^+/+^; Red: *Lmna* ^-/-^) in histogram indicate the percent of SP cells that are TCRβ^+^. F. CD4 SP and CD8 SP gated thymocytes were analyzed for the percent that are post-positive selection TCRβ+ mature SP thymocytes. Charts show the mean percent with error bars indicating the standard deviation (*Lmna^+/+^* N = 3; *Lmna^-/-^* N = 3 for each indicated age group). P values were calculated using an unpaired two-tailed Student's t test (* p<0.05, ** p<0.001, *** p<0.0001).

Given the striking decrease in thymic cellularity in *Lmna* ^-/-^ mice, we predicted a decrease in the proportion of the predominant CD4^+^CD8^+^ double-positive (DP) thymocyte population. Surprisingly, flow cytometric analysis revealed no perturbation in the frequency of DP thymocytes (Figure 2A and 2B). Slight reductions were noted in the percent of CD4 single-positive (SP) and CD8 SP thymocytes, starting at 4 weeks of age. There was also a significant increase in the proportion of double-negative (DN) thymocytes at 9 weeks of age in *Lmna* ^-/-^ mice, possibly indicating that cells are partially blocked in their developmental progress at one of the DN stages. To further explore this possibility, we analyzed DN thymocytes, and found that the percent of DN cells that were DN1 (least mature), DN2, DN3, and DN4 (most mature) remained unaltered in *Lmna* ^-/-^ mice, indicating that the relative increase in the percent of DN thymocytes at 9 weeks does not result from a developmental block at one of the DN stages (Figure S2).

After completion of TCRβ rearrangement in the thymus, cells proliferate at the DN3 to DN4 stage and upregulate CD4 and CD8 coreceptors to become CD4^+^CD8^+^ DP thymocytes. At the DP thymocyte stage, cells undergo TCRα rearrangement, and test the newly generated TCRα chain paired with the already available TCRβ chain for the ability to interact with self-MHC class I and class II molecules. Useful TCR interactions will result in positive selection of the cell, upregulation of TCR and CD69 surface expression, and lineage commitment of CD4 SP and CD8 SP thymocytes. Failure to be selected results in additional TCRα rearrangement, and eventually, if no positive selection takes place, the cell undergoes apoptosis (for review, see Starr et al.)[17]. We observed that the DPdull (CD4^low^CD8^low^) thymocytes, a sub-population of DP thymocytes that are normally enriched for selected TCRβ^+^ cells, lacked positively-selected TCRβ^high^CD69^+^ cells in 9-week-old *Lmna* ^-/-^ mice (Figure 2C and 2D). Although there was not a significant decrease in the percent of CD4 SP and CD8 SP gated thymocytes from 9-week-old *Lmna* ^-/-^ mice, these cells displayed clear signs of altered development, including decreased cell surface TCRβ expression for both CD4 SP and CD8 SP thymocytes, and decreased frequency of post-positive selection TCRβ^+^ mature CD8 SP thymocytes (Figure 2E and 2F). Thus, the development of TCRβ^+^ mature SP thymocytes is defective in *Lmna* ^-/-^ mice, suggesting that positive selection is impaired.

### Age-dependent progressive T and B cell lymphopenia in *Lmna* ^-/-^ mice

**Figure 3:**
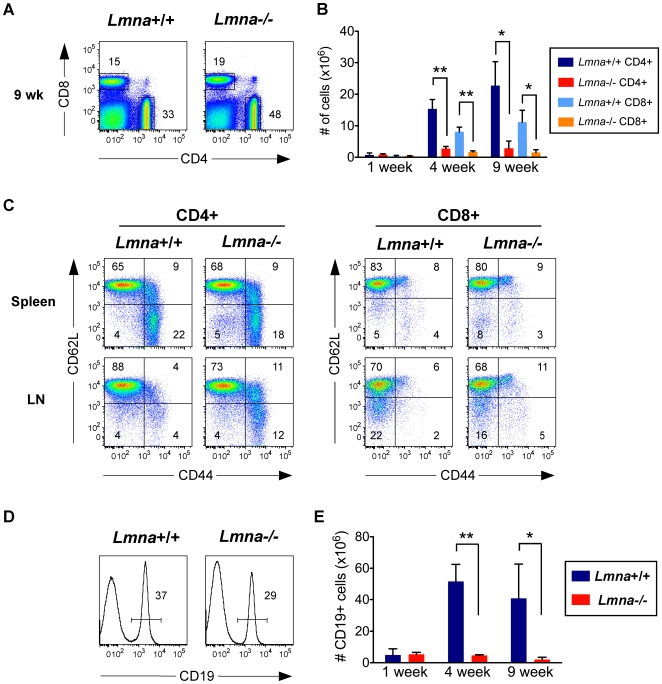
T cell and B cell numbers show an age-dependent decline in the lymphoid periphery of *Lmna^-/-^* mice. Splenocytes were surface stained for CD4, CD8, CD19, CD62L, CD44, and panTCRβ. A. Representative flow cytometry plots show the percent of CD4^+^ and CD8^+^ T cells. B. Absolute numbers of splenic CD4^+^ and CD8^+^ T cells for each indicated age group. C. Gated CD4^+^ and CD8^+^ splenocytes were analyzed for CD62L and CD44 expression. D. Representative histograms showing the percent of CD19^+^ B cells in the spleen. E. Absolute numbers of CD19^+^ B cells per spleen compiled for each indicated age group. Numbers in plots and histograms indicate the percentage of each gated population. Charts show the mean cell number with error bars indicating the standard deviation (*Lmna^+/+^* N = 3; *Lmna^-/-^* N = 3 for each indicated age group). P values were calculated using an unpaired two-tailed Student's t test (* p<0.05, ** p<0.001).

The apparent defect in positive selection in the thymus, compounded with the reduced thymic size in *Lmna^-/-^* mice, suggested the size of the peripheral T cell compartment in these mice would be diminished. To investigate this possibility, we analyzed splenic CD4^+^ and CD8^+^ T cells. While *Lmna* ^-/-^ mice exhibit an increased percent of splenic CD4^+^ and CD8^+^ T cells compared to *Lmna* ^+/+^ mice (Figure 3A), the absolute number of CD4^+^ and CD8^+^ T cells is dramatically decreased in *Lmna* ^-/-^ mice at both 4 and 9 weeks of age (Figure 3B). These reduced T cell numbers are a reflection of the loss in absolute numbers of total splenocytes in the *Lmna^-/-^* mice at these ages (Figure 1D). Given this severe T cell lymphopenia, we predicted that the remaining T cells in *Lmna^-/-^* mice would be driven to an activated/memory CD62L^low^CD44^high^ phenotype through lymphopenia-induced homeostatic proliferation. Surprisingly, there was no change in the percent of CD4^+^ and CD8^+^ cells that bear an activated/memory phenotype in 4-week-old (not shown) and 9-week-old *Lmna* ^-/-^ spleens (Figure 3C). Although there were subtle increases in the proportion of T cells that are CD44^high^ and either CD62^low^ or CD62L^high^ in the lymph nodes, these remained dramatically lower than other mouse systems characterized by T cell lymphopenia [18], [19]. In 9-week-old *Lmna* ^-/-^ spleens, we also observed an alteration in the percent of B cells (Figure 3D). This alteration coincided with a severe reduction in B cell numbers in the spleen (Figure 3E). Taken together, these data indicate that the development and/or survival of both B and T lymphocytes is defective in *Lmna* ^-/-^ mice.

### B cell development is interrupted in *Lmna^-/-^* mice

**Figure 4:**
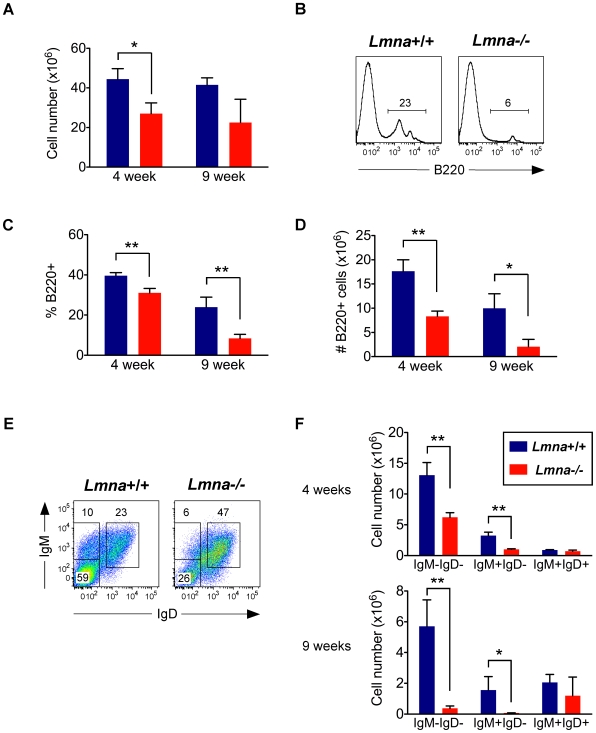
B cell development is impaired in *Lmna* ^-/-^ mice. Bone marrow single-cell suspensions were stained for surface B220, IgM, and IgD expression. A. Absolute number of bone marrow cells. B. Representative histograms from 9-week-old mice showing the percent of bone marrow cells that are developing B220^+^ cells. C. The percent of bone marrow cells that are B220^+^ for each indicated age group. D. Absolute number of B220^+^ bone marrow cells for each indicated age group. E. Representative IgM/IgD plots of gated B220^+^ bone marrow cells from 9-week-old mice. F. Absolute number of B220^+^ bone marrow cells that are IgM^-^IgD^-^, IgM^+^IgD^-^ (immature B cells), and IgM^+^IgD^+^ (mature B cells). Numbers in plots and histograms indicate the percentage of each gated population. Charts show the mean value with error bars indicating the standard deviation (*Lmna^+/+^* N = 3; *Lmna^-/-^* N = 3 for each indicated age group). P values were calculated using an unpaired two-tailed Student's t test (* p<0.05, ** p<0.001).

The cellularity of bone marrow from *Lmna* ^-/-^ mice was decreased in 4-week-old mice (Figure 4A). Because splenic B cell numbers were dramatically reduced in 4- and 9-week-old *Lmna* ^-/-^ mice compared to *Lmna* ^+/+^ mice, we analyzed bone marrow to determine whether B cell development was altered. Indeed, the population of developing B220^+^ cells committed to the B cell lineage was severely reduced in *Lmna^-/-^* mice, and composed primarily of a B220^high^ subpopulation (Figure 4B). This reduction occurred as early as 4 weeks of age in *Lmna* ^-/-^ mice (Figure 4C and 4D). Of the remaining B220^+^ cells in the bone marrow of *Lmna* ^-/-^ mice, there was a reduced percent and number of IgM^-^IgD^-^ cells and IgM^+^IgD^-^ immature B cells, and an increased percent but normal number of IgM^+^IgD^+^ mature B cells (Figure 4E and 4F). Because the developing B220^low^ IgM^-^IgD^-^ cells and IgM^+^IgD^-^ immature B cells are so severely affected in *Lmna* ^-/-^ mice, it is likely that these B220^high^ IgM^+^IgD^+^ mature B cells reached maturity before these mice experienced the precipitous decline in B cell development. Therefore, B cell development is impaired in *Lmna* ^-/-^ mice with age.

### *Lmna^-/-^* B and T cell defects are not cell-autonomous

**Figure 5:**
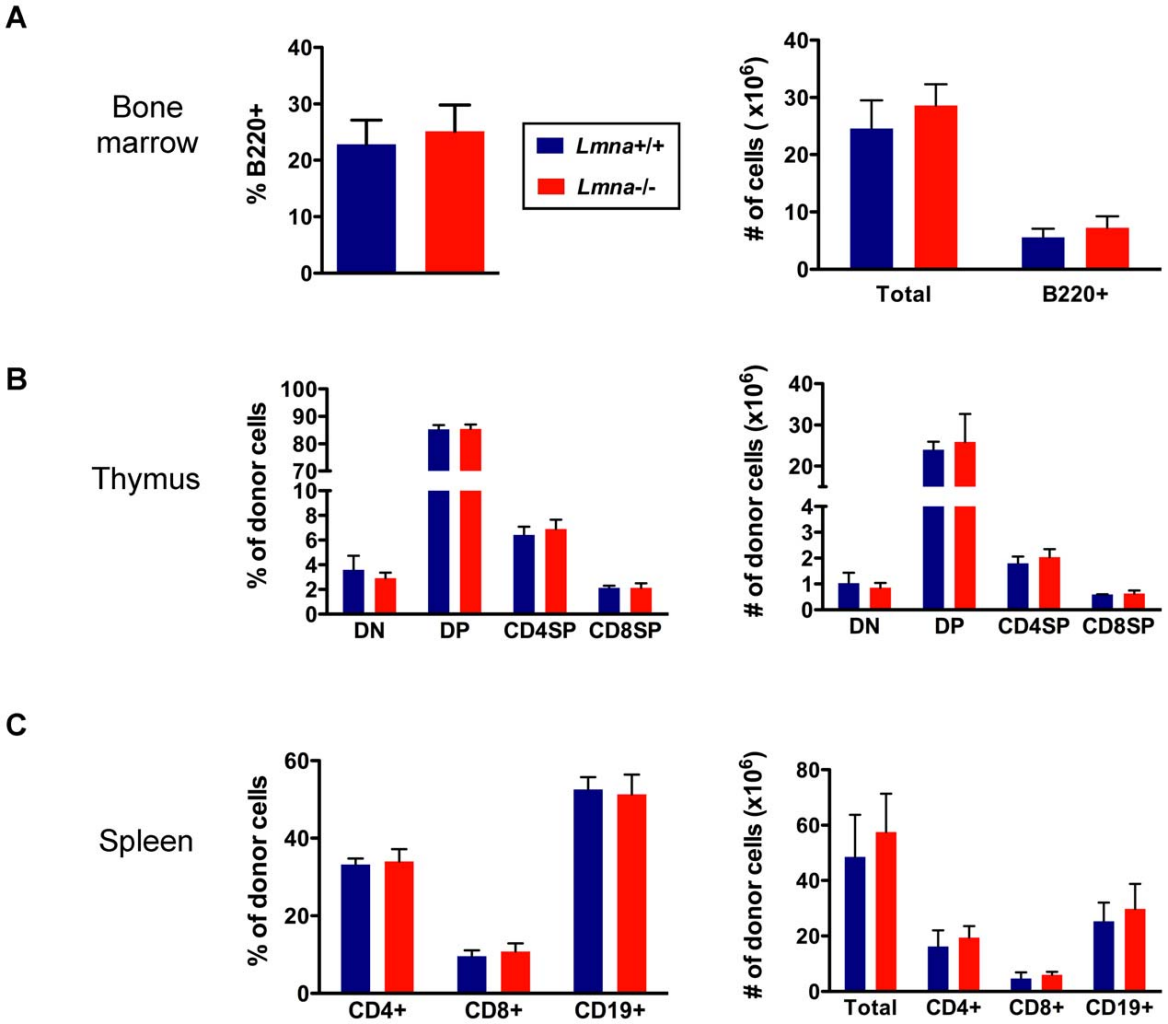
*Lmna* ^-/-^ T and B cell defects are not cell-autonomous. Bone marrow chimeras were generated by reconstituting irradiated wild-type Ly5.1^+^Ly5.2^+^ mice with bone marrow cells from either *Lmna* ^+/+^ or *Lmna* ^-/-^ Ly5.2^+^Ly5.1^-^ donor mice. Single-cell suspensions from bone marrow, thymus, and spleen were analyzed 44 weeks following irradiation and bone marrow reconstitution. Cells were stained for surface Ly5.1, Ly5.2, B220, CD4, CD8, TCRβ, and CD19 expression. Donor-derived Ly5.2^+^Ly5.1^-^ cells were analyzed in thymus and spleen. Total bone marrow cells were analyzed because not all bone marrow cells express Ly5; however, >98% of Ly5^+^ cells were donor-derived Ly5.2^+^Ly5.1^-^, indicating efficient engraftment. A. The percentage of total bone marrow cells that are B220^+^ (left) and the absolute number of total bone marrow cells and B220^+^ bone marrow cells (right) in the indicated bone marrow chimeric mice. B. The percent (left) and absolute number (right) of the indicated donor cells that are DN, DP, CD4 SP, and CD8 SP thymocytes. C. The percent (left) and absolute number (right) of the indicated donor cells that are CD4^+^ T cells, CD8^+^ T cells, and CD19^+^ B cells. Charts show the mean percent or number with error bars indicating the standard deviation (*Lmna* ^+/+^ into wild-type (WT) chimera N = 3; *Lmna* ^-/-^ into WT chimera N = 5). P values were calculated using an unpaired two-tailed Student's t test and none reached significance.

The alteration in lymphocyte development and homeostasis in *Lmna* ^-/-^ mice could result from cell-intrinsic defects caused by *Lmna* gene deficiency, from the contribution of A-type lamin function in non-hematopoietic cells that impact lymphocyte development, or instead, from the severe pathology and poor health of the *Lmna* ^-/-^ mouse. We generated bone marrow chimeras by reconstituting lethally irradiated *Lmna* ^+/+^ mice with *Lmna* ^+/+^ or *Lmna* ^-/-^ bone marrow to determine whether the defects in T and B cell development and homeostasis were cell-autonomous. Because thymocyte and splenocyte defects in *Lmna* ^-/-^ mice were age-dependent, we waited >40 weeks after irradiation and reconstitution to analyze the number of *Lmna* ^+/+^ and *Lmna* ^-/-^ populations in bone marrow, thymus, and spleen of chimeras. Bone marrow chimeric mice reconstituted with either *Lmna* ^-/-^ or *Lmna* ^+/+^ bone marrow had similar frequencies and numbers of total bone marrow cells and B220^+^ bone marrow cells (Figure 5A). Bone marrow chimeras that received either *Lmna* ^-/-^ or *Lmna* ^+/+^ bone marrow had identical relative and absolute numbers of donor-derived DN, DP, and SP thymocyte populations (Figure 5B). Additionally, the relative and absolute number of donor-derived B and T cells in the spleen were the same in chimeric mice reconstituted with either *Lmna* ^-/-^ or *Lmna* ^+/+^ bone marrow (Figure 5C).

**Figure 6:**
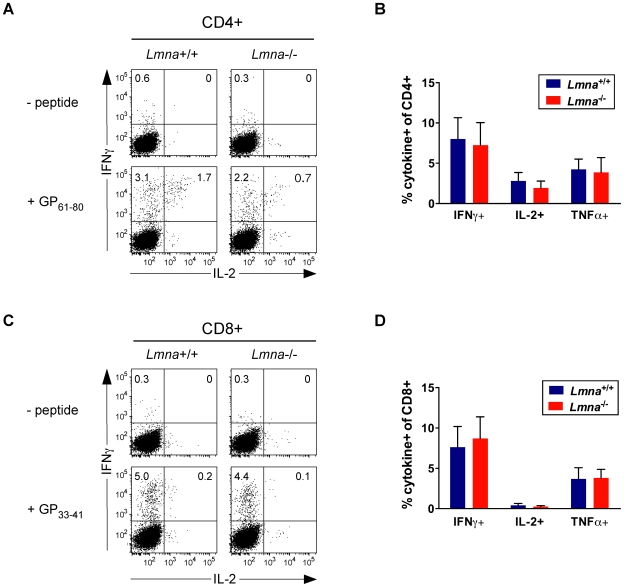
*Lmna* ^-/-^ T cells in mixed chimeric mice generate functional antigen-specific responses to viral infection. Mixed bone marrow chimeras were generated by reconstituting irradiated Ly5.1^+^Ly5.2^+^ mice with a mix of bone marrow cells from Ly5.1^+^Ly5.2^−^ *Lmna* ^+/+^ and Ly5.2+Ly5.1- *Lmna* ^-/-^ donor mice. Chimeric mice were infected with LCMV. Eight days later, splenocytes were restimulated with the relevant LCMV peptides for 5 hours, stained for cell surface CD4, CD8, Ly5.1, and Ly5.2 expression, and then stained for intracellular IFNγ and IL-2 or TNFα expression. Flow cytometric analysis allowed for clear separation of *Lmna* ^+/+^ (Ly5.1^+^Ly5.2^-^) and *Lmna* ^-/-^ (Ly5.1^-^Ly5.2^+^) donor T cells. A. Representative plots of intracellular IFNγ and IL-2 staining of *Lmna* ^+/+^ and *Lmna* ^-/-^ CD4^+^ cells within the same mixed bone marrow chimeric mouse. Antigen-specific CD4^+^ T cells produce cytokine when incubated in the presence of GP_61–80_ peptide (bottom plots) but not in its absence (top plots). B. The percent LCMV GP_61–80_-specific cytokine producing cells of donor-derived CD4^+^ T cells. C. Representative plots of intracellular IFNγ and IL-2 staining of *Lmna* ^+/+^ and *Lmna* ^-/-^ CD8^+^ cells within the same mixed bone marrow chimeric mouse. Antigen-specific CD8^+^ T cells produce cytokine when incubated in the presence of GP_33–41_ peptide (bottom plots) but not in its absence (top plots). D. The percent LCMV GP_33–41_-specific cytokine producing cells of donor-derived CD8^+^ T cells. Numbers in plots indicate the percent of each gated population. Charts show the mean percent with error bars indicating the standard deviation (*Lmna* ^+/+^/*Lmna* ^-/-^ mixed bone marrow chimeras N = 5). P values were calculated using an unpaired two-tailed Student's t test and none reached significance.

To assess the immunocompetence of *Lmna* ^-/-^ CD4^+^ and CD8^+^ T cells that appear to develop normally in *Lmna* ^+/+^ bone marrow chimeric hosts, we generated *Lmna* ^+/+^/*Lmna* ^-/-^ mixed bone marrow chimeras by reconstituting lethally-irradiated *Lmna* ^+/+^ mice with a mix of *Lmna* ^+/+^ and *Lmna* ^-/-^ bone marrow. Chimeras were infected 18 weeks post-reconstitution with the acute virus LCMV, known to generate a strong virus-specific response from both CD4^+^ and CD8^+^ T cells in B6 mice. Eight days later (at the peak of the T cell response), we analyzed *Lmna* ^+/+^ and *Lmna* ^-/-^ CD4^+^ and CD8^+^ T cells for the presence of antigen-specific effector T cells. IFNγ^+^, IL-2^+^, and TNFα^+^ virus-specific effector cells were present at the same frequencies among both *Lmna* ^+/+^ and *Lmna* ^-/-^ CD4^+^ T cells within the same mixed bone marrow chimera (Figure 6A and 6B). Similarly, virus-specific cytokine-producing effectors were present at the same frequencies among *Lmna* ^+/+^ and *Lmna* ^-/-^ CD8^+^ T cells (Figure 6C and 6D). These data show that *Lmna* ^-/-^ CD4^+^ and CD8^+^ T cells that develop in an A-type-lamin-sufficient environment can recognize foreign antigen in the context of self MHC, and proliferate and produce cytokine following antigen encounter. Therefore, in a *Lmna* ^+/+^ host, *Lmna* ^-/-^ T cells are appropriately selected and functional, even when in direct competition with *Lmna* ^+/+^ T cells.

Thus, the splenic and thymic cellularity defects and the apparent deficiencies in lymphocyte development observed in *Lmna* ^-/-^ mice are not cell-autonomous for T or B lymphocytes. This implies that A-type lamin function/expression is not required in these cell types for normal development and homeostasis. Further, it implies the possibility that A-type lamin expression in non-hematopoietic cells influences the function and development of these lymphocytes.

### *Lmna* ^-/-^ thymus supports normal T cell development

**Figure 7:**
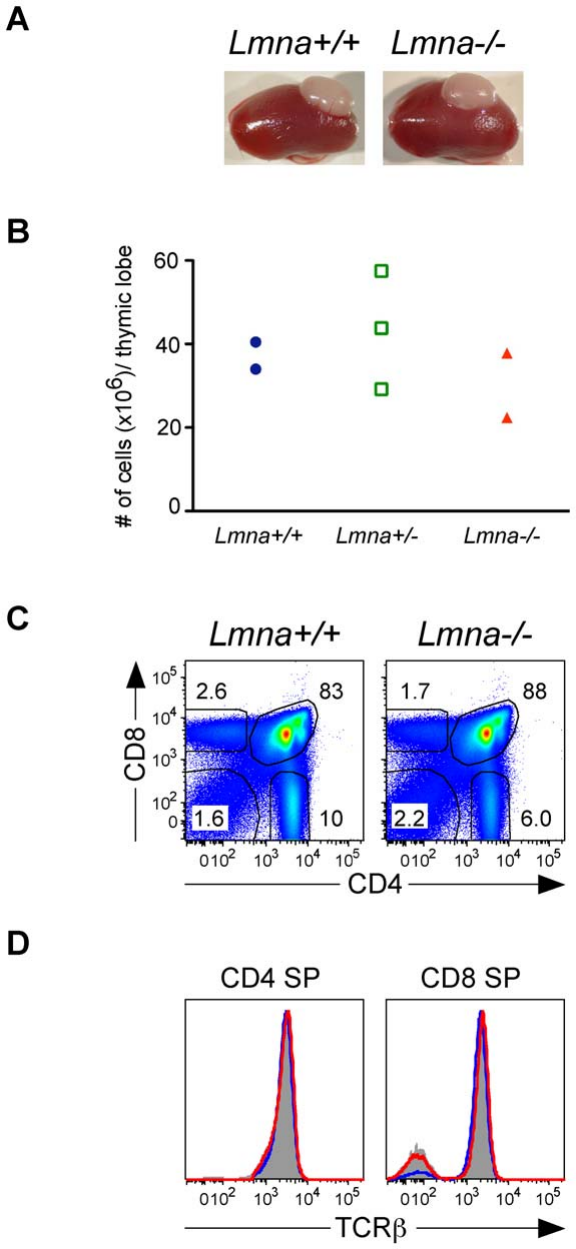
*Lmna* ^-/-^ thymic lobes support normal T cell development in *Lmna* ^+/+^ recipients. Thymic lobes from one-day-old *Lmna* ^+/+^, *Lmna* ^+/-^, and *Lmna* ^-/-^ neonates were transplanted under the kidney capsule of syngenic wild-type recipient mice. Thymic grafts were analyzed 6 weeks later, allowing sufficient time for seeding by stem cells from the *Lmna* ^+/+^ recipient, promoting subsequent thymocyte development within the grafted thymus. A. Kidneys of wild-type recipient mice with grafted *Lmna* ^+/+^ and *Lmna* ^-/-^ thymic lobes. B. The absolute number of thymocytes within the grafted thymic lobes. C. Representative flow cytometry plots show CD4/CD8 analysis of live-gated thymocytes from grafted *Lmna* ^+/+^ and *Lmna* ^-/-^ thymic lobes. D. Representative histogram overlays of gated CD4 SP and CD8 SP cells comparing TCRβ expression by *Lmna* ^+/+^ thymocytes (gray filled histogram), thymocytes from the *Lmna* ^+/+^ graft (blue histogram), and thymocytes from the *Lmna* ^-/-^ graft (red histogram). Data are compiled from two independent experiments.

We examined the possibility that defective T cell development in *Lmna* ^-/-^ mice might result from specific defects in thymic stromal cells, which are required for positive selection of developing DP thymocytes. To determine whether *Lmna* ^-/-^ thymic lobes could foster proper thymocyte development, we transplanted *Lmna* ^-/-^ neonatal thymic lobes into adult syngeneic recipients and analyzed thymic grafts 6 weeks later, after seeding of the lobes with host-derived *Lmna^+/+^* stem cells. Grafted *Lmna* ^-/-^ and *Lmna* ^+/+^ thymuses were similar in appearance and contained similar numbers of thymocytes (Figure 7A and 7B). Engraftment of both *Lmna* ^+/+^ and *Lmna* ^-/-^ thymic lobes appeared normal, as both supported a normal distribution of DN, DP, and CD4 SP and CD8 SP developing thymocytes (Figure 7C). Unlike the CD4 SP and CD8 SP thymocytes from *Lmna* ^-/-^ mice (see Figure 2E), CD4 SP and CD8 SP thymocytes within *Lmna* ^-/-^ thymuses grafted into *Lmna* ^+/+^ hosts had normal TCRβ expression and a similar percent of TCRβ+ mature SP thymocytes (Figure 7D). Therefore, *Lmna* ^-/-^ thymic stromal cells support normal T cell development when they are in a healthy, A-type-lamin-sufficient host.

## Discussion

In this study, we sought to determine the nature of lymphoid organ atrophy and lymphocyte population deficiencies in *Lmna* ^-/-^ mice. Young adult *Lmna* ^-/-^ mice exhibit striking decreases in splenic T and B lymphocyte numbers that correlate with reduced populations of developing thymocytes and B220^+^ B cells in bone marrow. The apparent selective block in T cell positive selection and early B cell development in *Lmna* ^-/-^ mice hinted at specific developmental checkpoints at which A-type lamin function was required. However, global reduction in both developing and mature lymphocytes coincided with the severe runting, as well as cardiac and skeletal muscle disease onset that occurs in *Lmna* ^-/-^ mice [7], [20]. In addition, 1-week-old neonatal *Lmna* ^-/-^ mice are healthy and indistinguishable from wild-type littermates, with comparable thymocyte numbers and splenic T and B cell numbers. These latter observations suggest a more general, perhaps indirect, impact on lymphocyte development resulting from the rapidly degrading health of older *Lmna* ^-/-^ mice.

Bone marrow chimeras were generated to test the possibility that lamin A/C expression is required in developing lymphocytes for normal T and B cell maturation. We did not find any evidence for this, as *Lmna* ^-/-^ bone marrow completely reconstituted wild-type recipient mice with normal T and B cell development and peripheral lymphocyte populations for more than 40 weeks following bone marrow transfer. *Lmna* ^-/-^ CD4^+^ and CD8^+^ T cell populations in mixed bone marrow chimeras were able to generate self-MHC class II and class I restricted T cell responses to LCMV infection. These antigen-specific and differentiated CD4^+^IFNγ^+^ and CD8^+^IFNγ^+^ effector cells were generated at the same frequency and demonstrated the same cytokine-producing potential from *Lmna* ^-/-^ and *Lmna* ^+/+^ cells within the same mixed bone marrow chimeras. These data show that even in differentiated T cell effector lineages, *Lmna* gene deficiency has no cell-autonomous consequence. Together, these data show that intrinsic lamin A/C expression is not required either for B cell development or for T cell development and function.

A second possible explanation for the lymphocyte developmental defects observed in *Lmna* ^-/-^ mice is that stromal elements within primary lymphoid organs require lamin A/C expression for their function in promoting normal T and B cell development. Thymic epithelial cells play a critical role in the positive selection of developing thymocytes as rearranged TCRα and TCRβ gene products are tested for useful and safe TCR interactions with self-MHC:peptide complexes. We tested whether there were thymic epithelial cell-specific defects in the *Lmna* ^-/-^ thymus by transplanting mutant thymic lobes into wild-type hosts, and allowing them to engraft for sufficient time to be seeded by wild-type hematopoietic stem cells. Within these mice, *Lmna* ^-/-^ thymic lobes maintained normal size and thymocyte cellularity compared to wild-type thymic grafts. Thus, *Lmna* ^-/-^ thymic stroma displayed no cell-intrinsic defects that can explain the reduced cellularity and altered thymocyte development observed in *Lmna* ^-/-^ mice.

However, a formal possibility still exists that defective stromal elements may influence B cell development in the *Lmna^-/-^* bone marrow. The stromal and hematopoietic microenvironments in the bone marrow are different than in the thymus and perhaps represent more complex interactions with diverse cell types. Of note, mesenchymal stem cells also inhabit the bone marrow and, in addition to their role in repopulating tissues of mesenchymal origin, have a regulatory role stimulating hematopoietic stem cell growth and differentiation (reviewed in Uccelli, et al.) [21], [22]. Because most tissues affected in laminopathy syndromes are of mesenchymal origin and because recent findings have suggested that altered A-type lamin function affects the maintenance of mesenchymal stem cell identity and ability to differentiate [23], defective communication between these cell types could also lead to altered lymphocyte development. Unfortunately, bone marrow architecture is difficult to preserve in transplantation studies, and future studies to address these possibilities will require conditional deletion of *Lmna* within select cell types of the bone marrow.

A third possible explanation for the lymphocyte developmental defects observed in *Lmna* ^-/-^ mice is an indirect effect of laminopathies at distal sites. *Lmna* ^-/-^ mice suffer from stunted growth and previously characterized cardiac and skeletal muscle defects contribute to their decline in health and premature death at around 6–8 weeks of age [7], [20], [24], [25]. Given that A-type lamins influence a variety of cell types, it is difficult to distinguish between cell-autonomous defects in specific tissues of *Lmna* deficiency and possible indirect effects rendered by so many dystrophic tissues. Thus, the atrophic lymphoid organs and accompanying reduced lymphocyte development and maintenance in the *Lmna* ^-/-^ mice may result from the overall poor health of the mice. These defects may be driven by altered stress hormone levels, cytokines from a chronic inflammatory response to striated muscle dysfunction that is present in *Lmna^-/-^* mice [7], [20], [26, Frock et al. manuscript in preparation], or decreased metabolic function resulting in nutritional deprivation of lymphocytes. Possibly supporting the argument for a role in nutritional defects, homeostasis of peripheral T cells appears to be impaired in *Lmna* ^-/-^ mice. Despite severe decreases in splenic T cell numbers, there was no upregulation of CD44 or downregulation of CD62L in spleen (Figure 3C), changes that characterize T cells that have undergone lymphopenia-induced homeostatic proliferation.

In summary, we have shown that *Lmna* ^-/-^ mice undergo a progressive decline in the size of their thymus and spleen, a decline of developing B220^+^ B cells in the bone marrow, a decline of total developing thymocytes, and striking decreases in peripheral B and T cells. All of these changes occurred after 1 week of age, and coincided with runting of *Lmna* ^-/-^ mice and onset of multi-tissue dystrophies. These immune defects were completely rescued when lymphocyte development was uncoupled from *Lmna* ^-/-^ animals through bone marrow chimera and thymic grafting experiments. We conclude that these effects on lymphocyte development in *Lmna* ^-/-^ mice are indirect, and most likely result from an unhealthy animal that is unable to support normal immune system development and homeostasis. Our data indicate that even apparently selective developmental blocks can arise from lymphocyte-extrinsic, indirect effects and underscore the need to analyze lymphocyte development in animals that provide a healthy environment before concluding that a particular gene or cell type of interest impacts development of a functional immune system.

## Supporting Information

Figure S1 Progressive cellularity defects in spleen and thymus occur even when organ cellularity is normalized to body weight of *Lmna^-/-^* mice. A. Thymic and B. splenic cellularity was normalized to the body weight of each individual mouse of each of the indicated ages. Charts show the mean normalized value with error bars indicating the standard deviation (*Lmna^+/+^* N = 3; *Lmna^-/-^* N = 3 for each indicated age group). P values were calculated using an unpaired two-tailed Student's t test (* p<0.05, ** p<0.001).
(0.25 MB TIF)

Figure S2 Normal composition of double-negative DN1-DN4 thymocytes in 9-week old *Lmna^-/-^* mice. Thymocytes from 9-week old mice were stained for CD4, CD8, CD25, and CD44 surface expression. DN thymocytes were analyzed for the percent that are DN1 (CD44^+^CD25^−^), DN2 (CD44^+^CD25^+^), DN3 (CD44^−^CD25^+^), and DN4 (CD44^−^CD25^−^). Chart shows the mean percent with error bars indicating the standard deviation (*Lmna^+/+^* N = 3, *Lmna^-/-^* N = 3). P values were calculated using an unpaired two-tailed Student's t test and none reached significance.
(0.10 MB TIF)
