# Supplementary figures and images for: Cell-Extrinsic Defective Lymphocyte Development in Lmna-/- Mice
Source: PLoS One. 2010 Apr 12;5(4):e10127. doi: 10.1371/journal.pone.0010127 (PMC2853576; doi:10.1371/journal.pone.0010127)

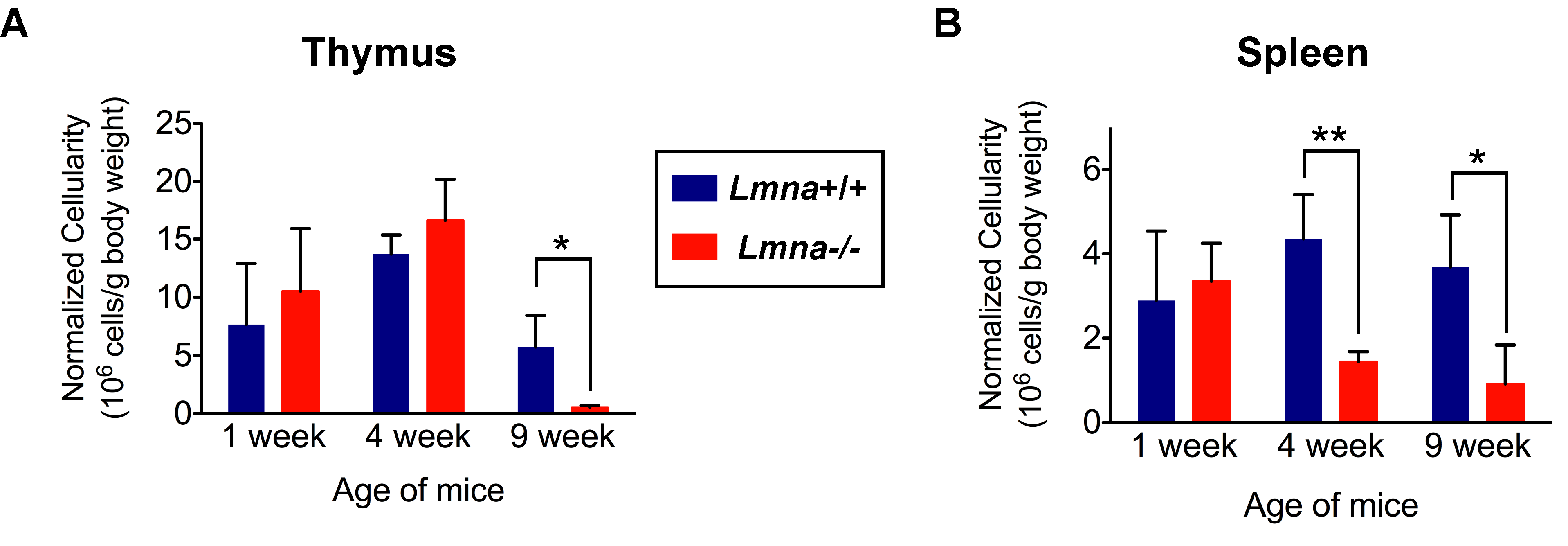

Supplement: Figure S1 — Progressive cellularity defects in spleen and thymus occur even when organ cellularity is normalized to body weight of Lmna-/- mice. A. Thymic and B. splenic cellularity was normalized to the body weight of each individual mouse of each of the indicated ages. Charts show the mean normalized value with error bars indicating the standard deviation (Lmna+/+ N = 3; Lmna-/- N = 3 for each indicated age group). P values were calculated using an unpaired two-tailed Student's t test (* p<0.05, ** p<0.001). (0.25 MB TIF) [file pone.0010127.s001.tif]

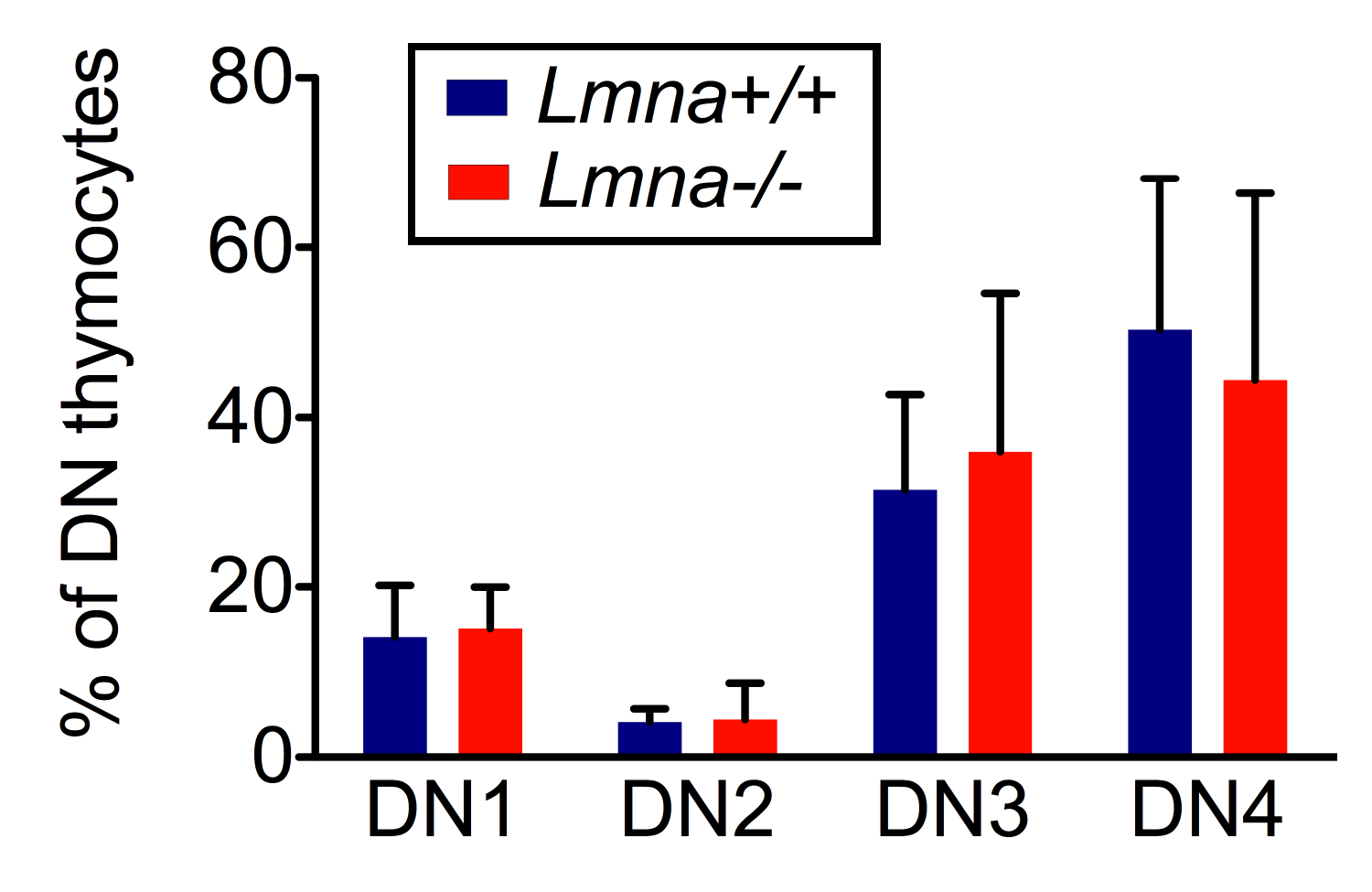

Supplement: Figure S2 — Normal composition of double-negative DN1-DN4 thymocytes in 9-week old Lmna-/- mice. Thymocytes from 9-week old mice were stained for CD4, CD8, CD25, and CD44 surface expression. DN thymocytes were analyzed for the percent that are DN1 (CD44+CD25−), DN2 (CD44+CD25+), DN3 (CD44−CD25+), and DN4 (CD44−CD25−). Chart shows the mean percent with error bars indicating the standard deviation (Lmna+/+ N = 3, Lmna-/- N = 3). P values were calculated using an unpaired two-tailed Student's t test and none reached significance. (0.10 MB TIF) [file pone.0010127.s002.tif]
